# Design of Pb(II)-Specific *E. coli*-Based Biosensors by Engineering Regulatory Proteins and Host Cells

**DOI:** 10.3389/fmicb.2022.881050

**Published:** 2022-05-20

**Authors:** Yangwon Jeon, Yejin Lee, Geupil Jang, Bong-Gyu Kim, Youngdae Yoon

**Affiliations:** ^1^Department of Environmental Health Science, Konkuk University, Seoul, South Korea; ^2^School of Biological Sciences and Technology, Chonnam National University, Gwangju, South Korea; ^3^Division of Environmental and Forest Science, Gyeongsang National University, Jinju, South Korea

**Keywords:** bacterial cell-based biosensors, heavy metals, Zn(II)-translocating P-type ATPase, protein engineering, Pb(II)

## Abstract

Bacterial cell-based biosensors have been widely developed for detecting environmental toxic materials. The *znt*-operon in *Escherichia coli* is a Zn(II)-responsive genetic system and is employed in Zn(II), Cd(II), and Hg(II)-sensing biosensors. In this study, point mutations were introduced in the regulatory protein ZntR to modulate its target selectivity, and metal ion-exporting genes, such as *copA* and *zntA*, in host cells were deleted to increase cellular metal ion levels and enhance specificity. Thus, the overall responses of the *E. coli* cell-based biosensors toward metal(loid) ions were increased, and their selectivity, which was originally for Cd(II) and Hg(II), was shifted to Pb(II). The gene encoding ZntA, known as the Zn(II)-translocating P-type ATPase, showed an impact on the ability of *E. coli* to export Pb(II), whereas *copA* deletion showed no significant impact. Noteworthily, the newly generated biosensors employing ZntR Cys115Ile showed the capacity to detect under 5 nM Pb(II) in solution, without response to other tested metal ions within 0–100 nM. To understand the marked effect of single point mutations on ZntR, computational modeling was employed. Although it did not provide clear answers, changes in the sequences of the metal-binding loops of ZntR modulated its transcriptional strength and target selectivity. In summary, the approaches proposed in this study can be valuable to generate new target-sensing biosensors with superior selectivity and specificity, which can in turn broaden the applicability of cell-based biosensors to monitor Pb(II) in environmental systems.

## Highlights

- The regulatory protein ZntR was mutated to modulate the target selectivity.
- Metal ion-exporting genes were deleted in host cells to improve specificity.
- The combination of these two elements led to enhanced detection potential toward Pb(II).

## Introduction

Lead (Pb) is a heavy metal widely used in diverse industries, including printing, construction, automobile manufacturing, rechargeable batteries, and others. Along with its widespread use, Pb has been released and is accumulated in environmental systems and is considered a risk for human health (Hernberg, 2000; Gould, 2009). It had been also reported that lead induces toxic stress in plants, resulting in the inhibition of seed germination and growth (Shu et al., 2012; Hadi and Aziz, 2015). Although leaded petrol and coal combustion have been the main contamination sources, it is also important to consider the effects of other potential contamination sources. Recently, shifts toward sustainable and green energy sources have occurred globally to protect the environment. In this regard, many companies are currently pursuing carbon neutrality, actively investing efforts into developing rechargeable batteries, which can be widely used in portable electronics and to replace internal combustion engines, especially, in vehicles. However, the massive production and usages of these batteries may also be potential sources of environmental pollutants such as diverse heavy metal(loid)s, including lithium, lead, and cobalt, among others (Nnorom and Osibanjo, 2009; Palacin, 2009; Yang et al., 2010; Placke et al., 2017). Thus, it is necessary to have tools for monitoring heavy metals from the wastes of rechargeable batteries and to recycle them.

Heavy metals in environmental systems, such as water, soil, and air, are typically analyzed by analytical instruments, including inductively coupled plasma-atomic spectroscopy and mass spectroscopy. Instrumental analysis shows high sensitivity and accuracy, but it can be time-consuming and expensive. Therefore, diverse metal sensing tools have been reported based on different techniques as potential alternatives to instrumental analysis approaches. Among these new techniques, bacterial cell-based biosensors have been actively investigated to quantify heavy metals (Magrisso et al., 2008; Kim et al., 2020a; Kannappan and Ramisetty, 2021). Importantly, bacterial cell-based biosensors are inexpensive and simple tools for fast detection of heavy metals, thereby overcoming the limitations of instrument-based analysis methods. Therefore, several target-detecting biosensors have been developed to monitor hazardous materials, including heavy metals, antibiotics, and chemicals, in diverse environmental systems (Elcin et al., 2021; Kannappan and Ramisetty, 2021; Shpigel et al., 2021; Zeng et al., 2021).

Recently, the targets of bacterial cell-based biosensors have been diversified upon findings of new genetic systems that respond to toxic materials, including formaldehyde, explosives, petroleum hydrocarbon, and nitrification inhibitors (Hynninen and Virta, 2009; Elcin et al., 2021; Henshke et al., 2021; Zappi et al., 2021). Owing to their simplicity and applicability, bacterial cell-based biosensors are considered alternative tools to monitor and assess risks for environmental pollutants. However, the number of available genetic systems is relatively limited compared with the number of pollutants resulting from industrial developments. Moreover, most genetic systems show a broad target specificity owing to the nature of living organisms, which may be disadvantageous. Hence, many researchers have made efforts to minimize the interference caused by nontarget materials. Noteworthily, enhancement of target specificity and selectivity was achieved by genetic engineering of regulatory proteins and host bacterial cells, rearrangement of genetic circuits, or by improving the detection techniques used (Nivens et al., 2004; Hynninen et al., 2010; Van Der Meer and Belkin, 2010; Changye et al., 2021).

In this study, we presented a strategy to generate target-specific biosensors using existing genetic systems. Briefly, by engineering the regulatory protein ZntR and deleting the metal ion-translocating P-type ATPases in host cells, a Pb(II)-specific biosensor was derived from Cd(II) sensing biosensors employing *znt*-operon as a genetic system as an alternative to the *pbr*-operon, which had been the only genetic system reported for Pb(II) detection (Rensing et al., 1998; Taghavi et al., 2009; Wei et al., 2014). Furthermore, we also demonstrated that by mutating ZntR, it is possible to achieve superior selectivity and specificity toward Pb(II) from the structural point of view. Hence, these novel strategies to generate biosensors may expand the targets for biosensors and thereby contribute to environmental monitoring and risk assessment of diverse pollutants.

## Materials and Methods

### Materials and *Escherichia coli* Strains

All heavy metal(loid)s used (e.g., AsCl_3_, CdCl_2_, CrSO_4_, HgCl_2_, PbSO_4_, MnCl_2_, AuCl, and ZnCl_2_) were purchased from Sigma-Aldrich (St. Louis, MO, USA). Novagen pCDF-Duet and pET-21(a) vectors (Sigma-Aldrich) were used to carry the sensing domain, *zntR* and mutants, and the reporter domain, *zntA* promoter fused with *egfp*, respectively (Yoon et al., 2016). *Pfu*Turbo DNA polymerase (Agilent, Santa Clara, CA, USA) was used for site-directed mutagenesis of *zntR*, and all restriction enzymes and T4 DNA ligase were purchased from Takara Korea Biomedical (Seoul, Korea). *E. coli* DH5α and BL21(DE3) strains were used for plasmid constructions and as host cells of the biosensors, respectively. *E. coli* strains, BL21-*copA/zntR* and BL21-*zntA/zntR* used in previous studies (Kang et al., 2018a; Kim et al., 2020b) and BL21-*copA/zntA/zntR*, newly generated in this study, were used as host cells for the biosensors. Endogenous gene deletion was performed using a Quick and Easy *E. coli* Gene Deletion Kit (Gene Bridges, Heidelberg, Germany). All strains used in the study are listed in Online Resource 1 with a description of their genetic properties.

### Endogenous Gene Deletion in *E. coli* BL21

Triple-gene-deficient *E. coli* was generated by deleting *copA* from BL21-*zntA/zntR* using a Quick & Easy *E. coli* Gene Deletion Kit. Briefly, the FRT-flanked PGK-gb2-neo cassette and *copA* sequences were amplified by a polymerase chain reaction and electroporated into *E. coli* BL21-*zntA/zntR* cells harboring the pRedET plasmid, which has the recombinase-encoding gene. Electroporation was performed with an Electroporator 2510 (Eppendorf, Hamburg, Germany) at 1,350 V, 10 μF, and 600 Ω (Kang et al., 2018b). The cells were then induced by adding 10% of arabinose to replace *copA* with the kanamycin resistance gene (*kan*). Gene deletion was further verified by polymerase chain reaction.

### Engineering of ZntR

Engineered ZntRs were generated by site-directed mutagenesis of the metal-binding loop (MBL) region, consisting of 12 amino acids. The nucleotide sequences for the amino acid residues surrounding the MBL were mutated to encode specific amino acids using designed primers, which was confirmed by DNA sequencing. The mutation targets were selected upon analysis of the three-dimensional structure of the ZntR. The engineered ZntRs used (Table 1) were tested as sensing domains for metal ion detection in *E. coli* BL21-*zntR*, BL21-*copA/zntR*, BL21-*zntA/zntR*, and BL21-*copA/zntA/zntR*. The primers for gene cloning and point mutations are listed in Online Resource 2.

**Table 1:** List of recombinant ZntRs tested and their responding orders toward heavy metals.

**No**.	**ZntRs**	**MBL sequences (aa: 114–125)^**a**^**	**Responding orders and strengths** ^**b**^	
			***E. coli-zntR***	***E. coli-zntR/copA/zntA***
1	ZntR WT	CCGTAHSSVYCS	Cd (3.1) > Pb (1.3) > Hg (1.2)	Pb (3.2) > Cd (2.8) > Hg (1.8)
2	ZntR C115I	C**I**GTAHSSVYCS	Pb > Cd > Hg	Pb (27.8) > Cd (12.2) > Hg (3.5)
3	ZntR C115S	C**S**GTAHSSVYCS	Pb > Cd > Hg	Pb (6.1) > Cd (4.7) > Hg (1.7)
4	ZntR T117del	CCG–AHSSVYCS	–	–
5	ZntR H119R	CCGTA**R**SSVYCS	Pb > Cd > Hg	Pb (7.5) > Cd (4.7) > Hg (1.5)
6	ZntR C124S	CCGTAHSSVY**S**S	–	–
7	ZntR C141S	CCGTAHSSVYCS	Pb > Cd > Hg	Pb (4.0) > Cd (2.4) > Hg (1.2)

a *Mutated amino acids are indicated in bold/underlined letters in the MBL sequences. Cys141 is not shown in the sequences because it was located at the C-terminus of the ZntR*.

b *Responding strengths toward heavy metals are indicated as the induction coefficient values obtained from metal selectivity assays with 1 μM exposure. The values are represented as mean values obtained from more than triplicate experiments, and errors are not shown.*

*The bold letters indicated the changes in amino acid sequences compared to ZntR WT*.

### Biosensor Assay

*Escherichia coli* cell-based biosensors were generated by introducing a pair of plasmids carrying *zntAp::egfp* and engineered *zntR* in genetically modified *E. coli* BL21 strains, including *E. coli-zntR, E. coli-zntR/copA, E. coli*-*zntR/zntA*, and *E. coli-zntR/copA/zntA*. The biosensor assay was conducted as previously described with some modifications (Kang et al., 2018b; Yoon et al., 2018). Briefly, the biosensor cells were grown at 37°C in a shaking incubator overnight, and 1 ml of the cultures was then inoculated in 50 ml of fresh Luria broth until it reached an OD_600_ value of 0.10. Next, the cells were placed in test tubes and directly exposed to metal ions. After 2 h of incubation, the *E. coli* cells were harvested, and the cell pellet was resuspended in Tris buffer (50 mM Tris-HCl, 160 mM KCl, pH 7.4) before measuring fluorescence intensity.

### Fluorescence Intensity Analysis

The intensity of the fluorescence signals generated by the biosensors after heavy metal exposure was measured using fluorescence spectroscopy (FC-2; Scinco, Seoul, Korea). The expression of the enhanced green fluorescent protein (eGFP) was measured at 480 and 510 nm for excitation and emission wavelength, respectively. The fluorescence signals induced by heavy metal exposure were represented as the induction coefficient values defined as (fluorescence intensity of metal-exposed biosensors)/(fluorescence intensity of non-exposed biosensors). The detection limit (DL) for biosensor assay was calculated by the following: DL = 3 s/*m* (s: standard deviation obtained from multiplicated measurements; *m*: slope of the standard curve).

### Structural Analysis

The crystal X-ray structure of ZntR was obtained from the Protein Data Bank (PDB ID:1Q08) and modified amino acids by PyMol (Schrödinger, New York, NY, USA). Sybyl 7.3 (Tripos, St. Louis, MO, USA) was used for energy minimization processing. In the protein structures, water molecules and phosphate ions were removed, and hydrogens were added. After modification, they were minimized using the Tripos force field. The energy minimization process was terminated at the convergence criteria of the total energy (0.05 kcal/molÅ). Chimera 1.11 software (https://www.cgl.ucsf.edu/chimera/download.html) was used for protein visualization and analysis.

### Data Analysis

R v4.1.0 (https://cran.r-project.org) and *DescTools* package were used for the statistical analysis of all data when the minimum difference between the control and treatment groups was statistically significant (Team, 2014; Signorell et al., 2019).

## Results

### Effects of Metal-Translocating ATPases on Metal Selectivity of Biosensors

**Figure 1:**
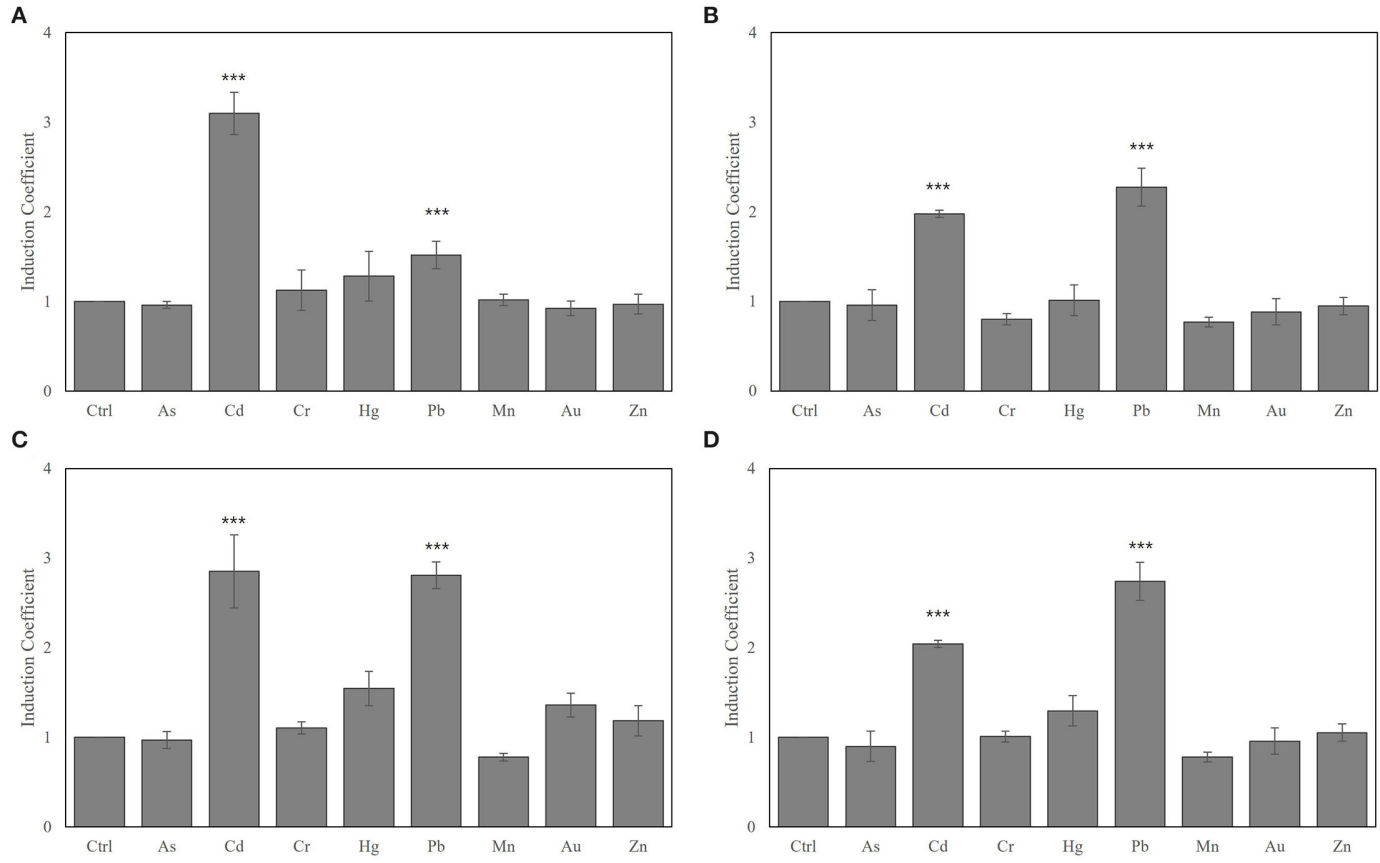
Metal selectivity of biosensors using **(A)** *E. coli-zntR*, **(B)** *E. coli-zntR/copA*, **(C)** *E. coli-zntR/zntA*, and **(D)** *E. coli-zntR/copA/zntA*. The biosensors were exposed to 1 μM of tested metal ions for 2 h, and the eGFP expression levels were represented as induction coefficient values. The values were obtained from more than three times of replicated experiments. The asterisk means the data were significantly higher than the control (Dunnett's test, ***p < 0.001).

Since biosensors based on *zntA-*deficient strains showed strong fluorescence signals even without metal exposure, the experimental conditions used in this study for heavy metal selectivity were modified from those previously reported (Yoon et al., 2016; Kang et al., 2018a). The metal selectivity of the biosensors using *E. coli-zntR, E. coli-zntR/zntA*, and *E. coli-zntR/copA/zntA* with wild-type (WT) ZntR was tested under optimized experimental conditions. Since further incubation was required before exposition to metal ions, the responses indicated as induction coefficient values and background fluorescent signals were decreased. Various metal ion concentrations (0.5, 1.0, 5.0, and 10 μM) were tested; the induction coefficient values induced by 1 μM metal ions are shown in Figure 1. As reported previously, *E. coli* WT-based biosensors showed Cd(II)-specific response although the induction coefficient values were decreased (Figure 1A; Yoon et al., 2016). Hence, the optimized experimental conditions were reliable to test the detection potential of the biosensors. Interestingly, Pb(II) responses were enhanced by *copA* or *zntA* deletions, whereas Cd(II) responses were slightly decreased (Figures 1B,C). Moreover, the order of strength was inversed as Pb(II) > Cd(II). In turn, the Pb(II) responses were clearly enhanced, and the responding order to heavy metals was changed to Pb(II) > Cd(II) > Hg(II) when both *copA* and *zntA* were deleted (Figure 1D). In addition, it was necessary to mention that the responses toward Zn(II) were not significant. Although the *znt*-operon had been identified as a Zn(II)-responsive genetic system, the regulatory protein, ZntR, prefers Cd(II) and Hg(II) rather than Zn(II). It was consistent with our previous data (Yoon et al., 2018). Thus, deleting *zntA* in *E. coli* led to increased Pb(II) responses, although the induction coefficient values varied. The effects of *copA* deletion on metal responses were less significant than those obtained upon *zntA* deletion, which suggests that *zntA* plays more relevant roles in divalent metal homeostasis in *E. coli* cells. In fact, it was unclear how metal-translocating channels affect the metal selectivity of *E. coli*-based biosensors. Nevertheless, metal selectivity and sensitivity can be modulated by disrupting metal homeostasis in the host cells.

### Effects of Genetic Engineering on ZntRs

Since the *E. coli* biosensors employed the *znt* operon, the target selectivity and sensitivity were determined by using ZntR, a known regulatory protein of the *znt* operon (Brocklehurst et al., 1999). Thus, the amino acids in the MBL regions of ZntR were selected as targets for genetic engineering to modulate its metal selectivity, and the engineered ZntRs were introduced into *E. coli-zntR/copA/zntA* strains with *zntAp::egfp* to generate the enhanced biosensors (Table 1).

**Figure 2:**
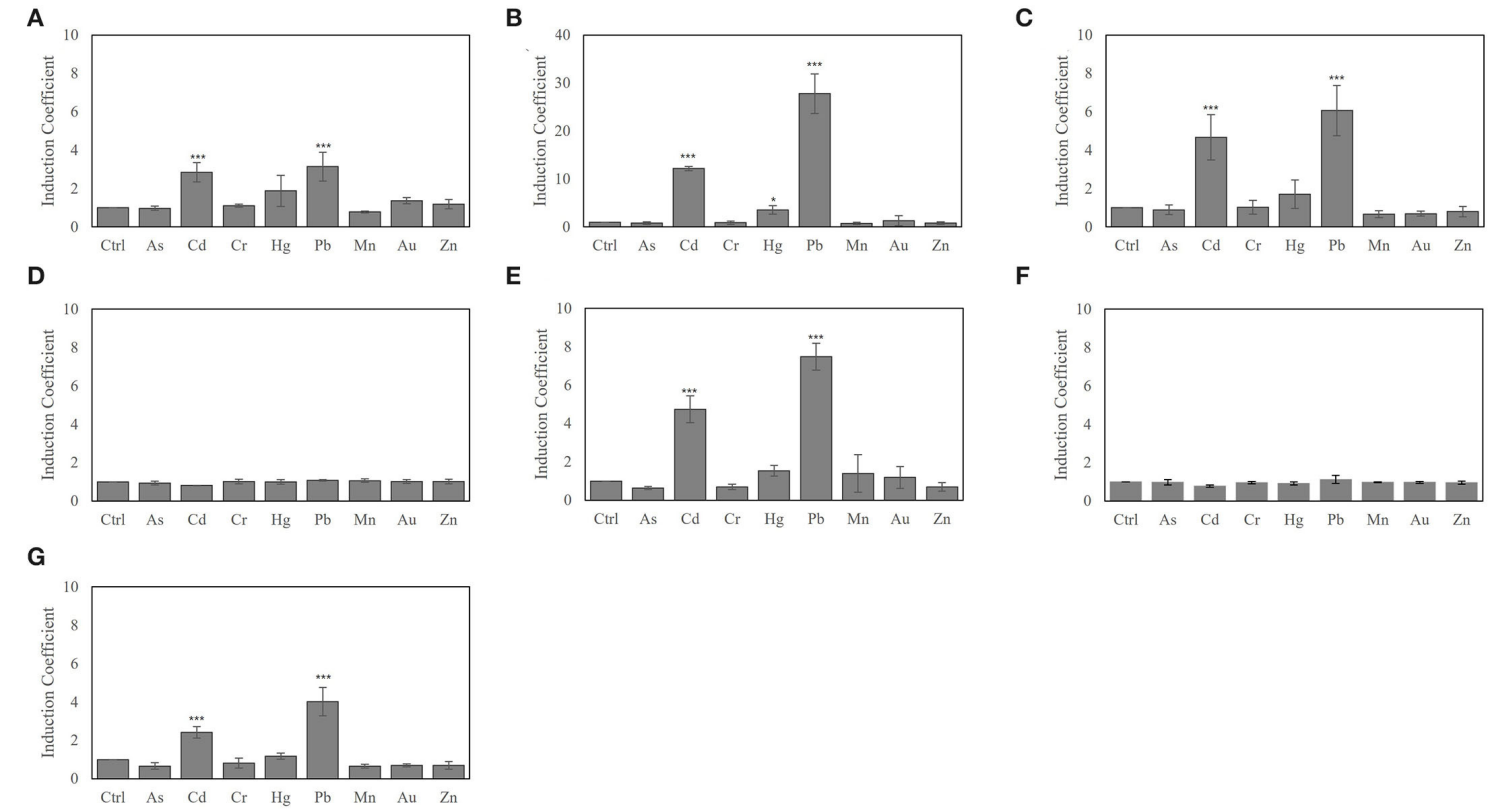
Modulating metal selectivity of biosensors based on *E. coli-zntR/copA/zntA* harboring pZnt-eGFP and ZntR WT **(A)**, C115I **(B)**, C115S **(C)**, T117del **(D)**, H119R **(E)**, C124S **(F)**, and C141S **(G)**. The biosensors were exposed to 1 μM of tested metal ions for 2 h, and the eGFP expression levels were represented as induction coefficient values. The asterisk means the data were significantly higher than the control (Dunnett's test, ***p < 0.001, *p < 0.005).

The biosensor cells were exposed to 1 μM of heavy metal ions, including As(III), Cd(II), Cr(II), Hg(II), Pb(II), Mn(II), Au(I), and Zn(II), and the fluorescence signals were measured after 2 h of exposure (Figure 2). Using ZntR WT as regulatory protein produced similar responses to Cd(II) and Pb(II), reaching ~3-fold induction coefficient values, and the same strength to Hg(II) and Au(I) as observed in the *E. coli-zntR/copA/zntA* strains (Figure 2A). Moreover, the orders of response strength to heavy metals were changed to Pb(II) > Cd(II) > Hg(II) in the systems engineered with mutated ZntR (Cys115Ile, Cys115Ser, His119Arg, and Cys141Ser) (Figure 2). Although it was unclear why the target selectivity of the biosensors shifted due to the point mutations, it was hypothesized that mutations on the metal-binding sites altered the ability of the cell to interact with the metal ions and induce conformational changes to selectively prefer Pb(II) over Cd(II). Since Cys115 and His119 directly mediate the conformational interaction with the metal ions, the selectivity of the engineered ZntRs was modulated by these amino acids. In addition, the Cys141 mutation, which is located in the C-terminal region of ZntR, also shifted the target selectivity from Cd(II) to Pb(II). Although this outcome was unexpected, the Cys141Ser mutation may induce conformational changes that affect metal selectivity. However, the biosensors employing ZntR Thr117 deletion and Cys124Ser lost their capacity to respond to the presence of the metal ions (Figures 2D,F).

Most interestingly, it was noticed that biosensors having ZntR Cys115Ile as a regulatory protein showed extremely strong responses to Pb(II) at induction coefficient values of over 35 (Figure 2B). Although it showed strong responses to Cd(II) and Hg(II) compared with ZntR WT, the response to Pb(II) was significant. In this regard, the *E. coli-zntR/copA/zntA* biosensors having ZntR C115I were investigated to further elucidate the potential as Pb(II)-specific biosensors and to understand the mechanisms of superior responses to Pb(II).

### Heavy Metal Sensitivity of Biosensors With Engineered ZntRs

**Figure 3:**
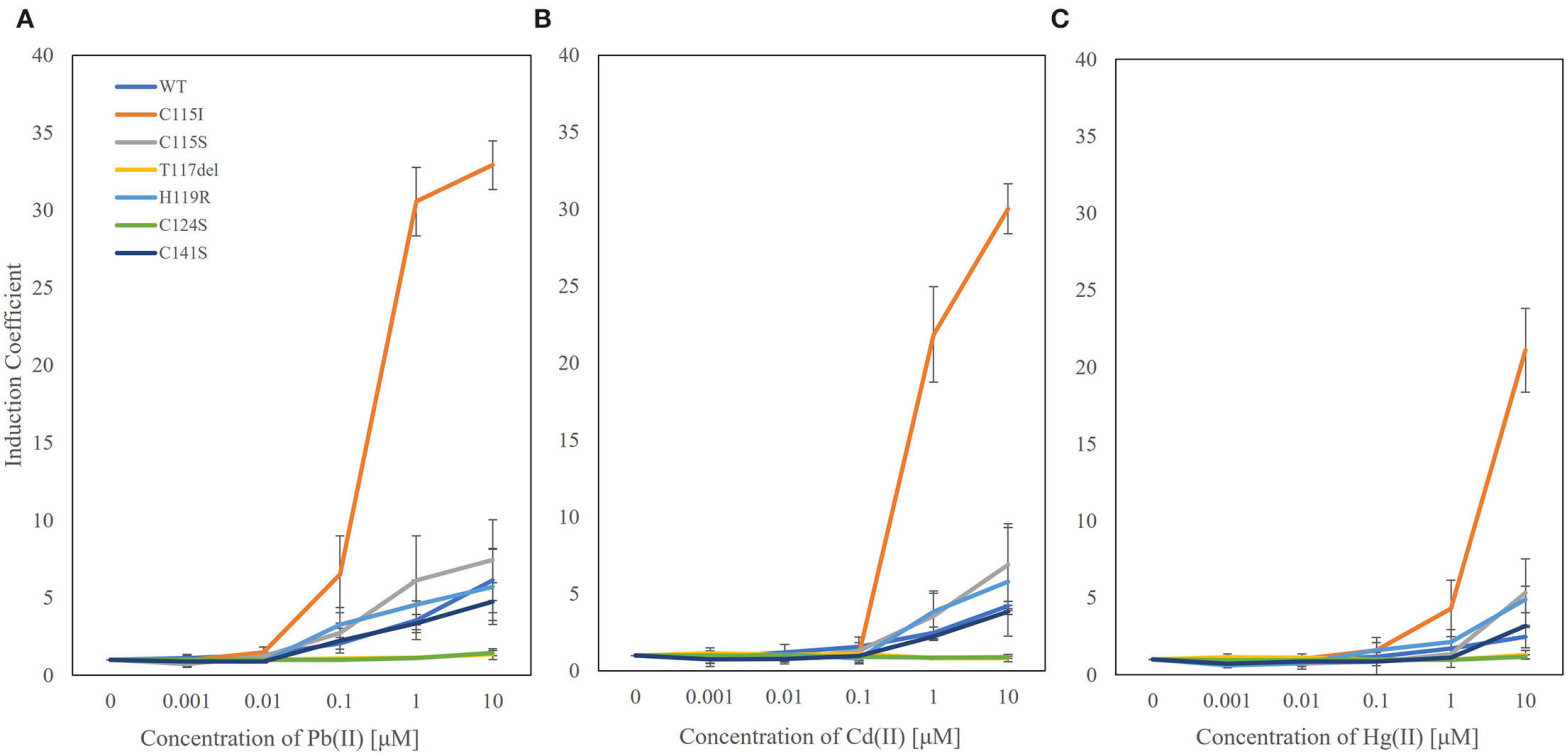
The metal specificity of biosensors based on *E. coli-zntR/copA/zntA* harboring pZnt-eGFP and ZntRs toward lead **(A)** cadmium **(B)** and mercury **(C)**. The biosensors were exposed to 0 to 10 μM ranges of metal ions and determined the induction coefficient values after 2 h exposures.

The biosensors generated with the engineered ZntRs and *E. coli-zntR/copA/zntA* showed modulated metal selectivity and responses to heavy metals (1 μM) upon 2 h of exposure. To elucidate the sensitivity to each heavy metal tested, the responses of the biosensors exposed to 0–10 μM of Pb(II), Cd(II), and Hg(II) were determined under the same experimental conditions (Figure 3). The biosensors employing ZntR WT, Cys115Ser, His119Arg, and Cys141Ser showed responses to all three tested metal ions in the order of induction coefficient values of Pb(II) ≈ Cd(II) > Hg(II). Noteworthy, the biosensor employing ZntR Cys115Ile showed superior signals toward Pb(II), Cd(II), and Hg(II); however, it had superior specificity toward Pb(II) over the other heavy metals at 0.1 μM and below (Figure 3). The induction coefficient value for Pb(II) was ~8, whereas it was less than 2 for Cd(II) and Hg(II). Thus, *E. coli-zntR/copA/zntA* biosensors with ZntR Cys115Ile as the regulatory protein may be Pb(II)-specific biosensors within certain concentration ranges.

### Characterization of Pb(II)-Specific Biosensor

**Figure 4:**
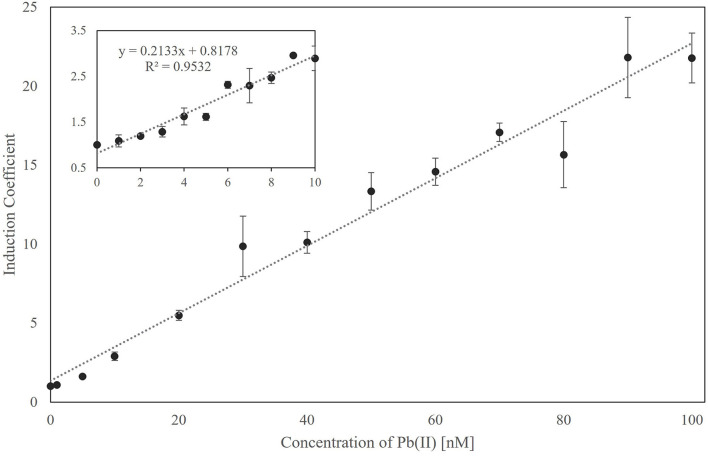
The relationship between the responses of biosensors employing ZntR-C115I as a regulatory protein and Pb(II) concentration ranged from 0 to 100 nM. The responses from 0 to 10 nM of Pb(II) were shown in the inbox, and the relationship was analyzed by a linear regression fit with 0.953 of *R*^2^ value.

Since the newly generated biosensor employing ZntR Cys115Ile showed responses toward Pb(II) only at 0.1 μM, the properties of the biosensors were further characterized. The responses of the biosensor to Pb(II) were determined at concentrations below 100 nM (Figure 4). Overall, the biosensor responded in a concentration-dependent manner, with its detection limit being calculated at ~5 nM Pb(II) and statistical significance being observed at 1 nM Pb(II) (Figure 4, inbox). Moreover, the relationship between the induction coefficient values and Pb(II) concentration showed a linear correlation, thereby allowing for quantitative analysis.

**Figure 5:**
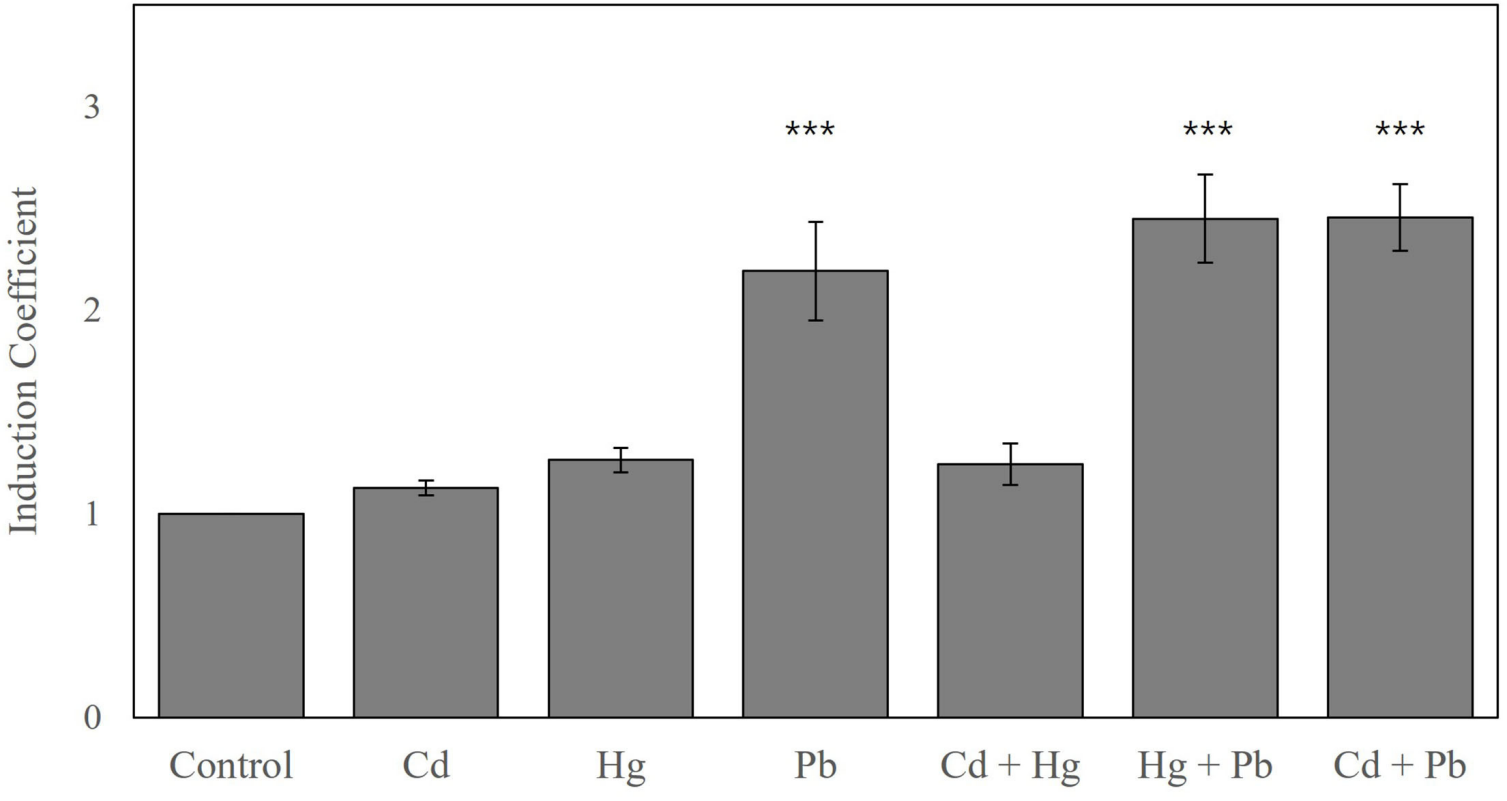
The comparative and combined exposures of biosensors *E. coli-zntR/copA/zntA* harboring pZnt-eGFP and ZntR C115I to Pb(II), Cd(II), and Hg(II). The biosensors were exposed to 10 nM of metal ions comparatively and combinedly, and the responses were represented as induction coefficient values. The asterisk means the data were significantly higher than the control (Dunnett's test, ***p < 0.001).

Interference of the other heavy metals, such as Cd(II) and Hg(II), on Pb(II) detection was also investigated to elucidate the robustness of the biosensor to serve as a Pb(II) detector. The biosensors were exposed to 10 nM of Pb(II), Cd(II), and Hg(II) alone or in combination (Figure 5). Biosensors exposed to Cd(II) and Hg(II) showed 0.12- and 0.25-fold increased induction coefficient values, respectively, whereas Pb(II) increased the fluorescence signal by 2.2-fold. When exposed to a combination of heavy metal ions, such as Cd(II)/Pb(II) or Hg(II)/Pb(II), the biosensor showed 2.4-fold induction coefficient values, which were comparable to those obtained with Pb(II) alone. Although the signals were slightly increased by Cd(II) and Hg(II) presence, the new biosensor was shown to have superior Pb(II) selectivity and specificity at certain concentration ranges.

### Structural Aspects of Engineered ZntRs

**Figure 6:**
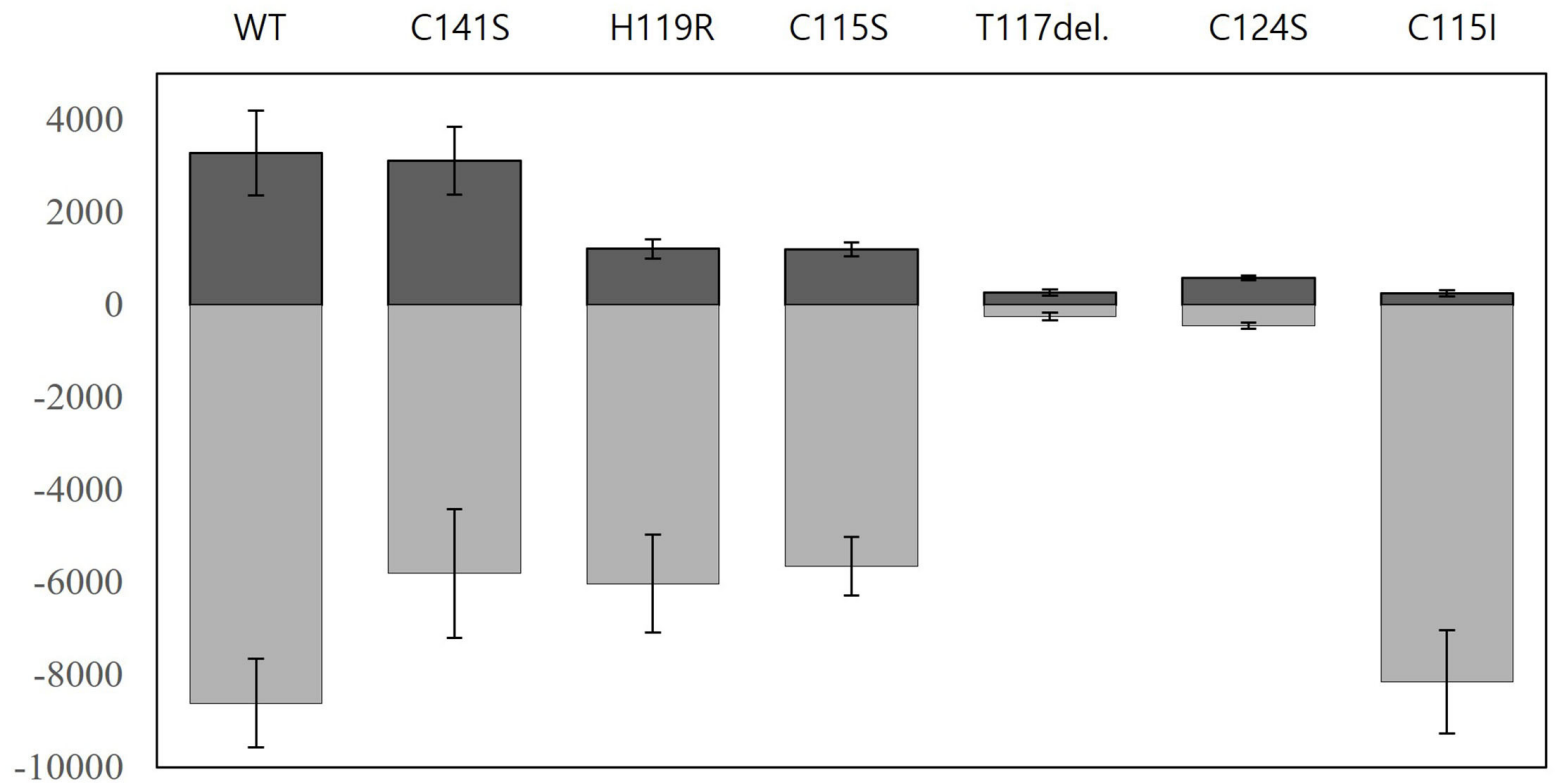
Nascent fluorescence signals of biosensors employing ZntR WT and mutants. The fluorescence intensities of biosensors based on *E. coli-zntR/copA/zntA* harboring pZntA-eGFP and ZntRs were measured after 2 h exposures to 0 μM and 1 μM of Pb(II). Positive (dark gray) and negative (light gray) values indicate the arbitrary units of eGFPs induced by non-exposure and exposure, respectively. The experiments were replicated more than three times and the standard deviations were indicated as error bars.

As described above, the engineered ZntR granted improved target selectivity and specificity to the biosensors while using *E. coli-zntR/copA/zntA* as host cells. To answer why and how the specificity and selectivity were modulated, it was necessary to analyze the fluorescence intensity, since the responses were determined as induction coefficient values. Hence, the fluorescence signals from Pb(II)-exposed and Pb(II)-non-exposed biosensors were screened (Figure 6). In the case of Thr117 deletion and Cys124Ser variant, the signals were similar regardless of the presence or absence of the metal ion, resulting in no increase of the induction coefficient value. Nonetheless, the other biosensors showed similar selectivity toward Pb(II), Cd(II), and Hg(II) although the induction coefficient values varied (Figure 2). As shown in Figure 6, the ZntR WT in *E. coli-zntR/copA/zntA* showed a strong signal even without Pb(II) exposure, which may be explained by the increased level of endogenous metal ions due to the deletion of the metal exporters that consequently turned on the *egfp* transcription. However, this assumption would not be accepted because the background signals of ZntR Cys15Ile were much lower even if they responded to metal ions better. In addition, the mutations on the MBL, such as His119Arg and Cys115Ser, showed less background and induced signals compared with ZntR WT in *E. coli-zntR/copA/zntA*. Furthermore, Cys141Ser located at the C-terminal region of ZntR showed no contribution for weaker background signals. Therefore, it was assumed that conformational changes on ZntRs were responsible for the variable strength observed to initiate the transcription of the reporter gene.

**Figure 7:**
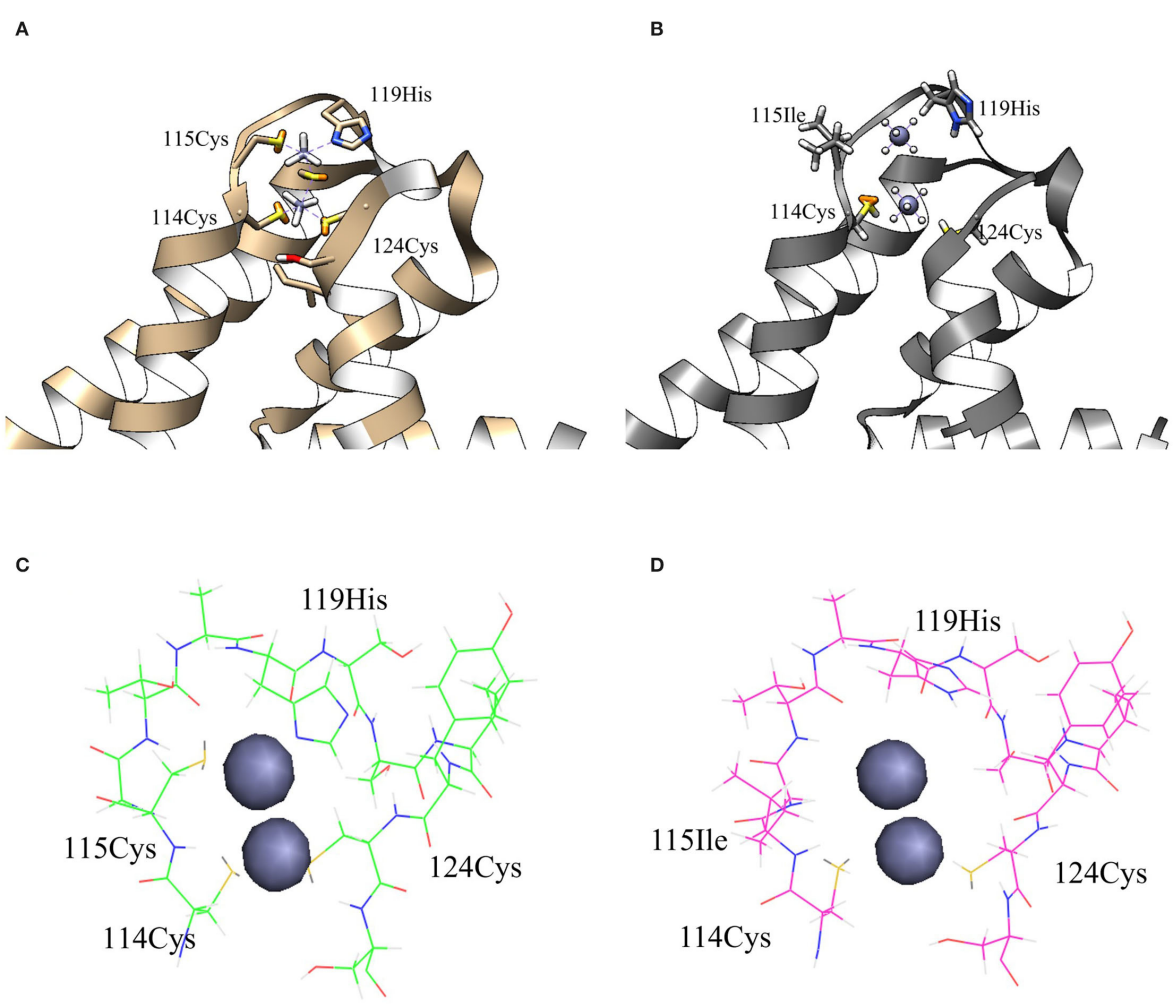
The structures of metal-binding loop regions for ZntR WT and C115I. The structures of MBLs for ZntR WT **(A)** and C115I **(B)** with stick representation of 4 residues interacting with zinc ions. The stick representation of amino acids consisted of MBLs for ZntR WT **(C)** and ZntR C115I **(D)**. The zinc-binding sites of wild type (green) and mutant of C115I (magenta) are shown as the stick representation.

To elucidate this assumption, structural analysis was performed by computational three-dimensional modeling and simulation. Overall, the MBL of ZntR C115I was relatively looser compared with that of the WT because of the hydrophobic nature of isoleucine (Figures 7A,B). It was clear when only MBLs were shown as stick representation that three cysteines and one histidine were placed closer to two zinc ions in the WT compared with those amino acids of the mutant C115I (Figures 7C,D). Additionally, the distances between the zinc ions and residues around the MBL, including three cysteines and one histidine, were measured (Table 2). Taken together, the enlargement of the metal-binding site may be the reason why ZntR Cys115Ile showed enhanced Pb(II) specificity. However, it was still unclear why the different mutations led to variable transcription levels. In fact, we expected that the mutations on the metal-binding site could lead to conformational changes of the ZntR DNA-binding domains, but no significant differences were observed in the model structures.

**Table 2:** Distances between the metal ions and residues located at the metal-binding loops of ZntR wild-type (WT) and Cys115Ile mutant measured from the model structures.

**ZntR WT**		**ZntR Cys115Ile**	
**Residue/metal ion**	**Distance (Å)**	**Residue/metal ion**	**Distance (Å)**
114Cys S–Zn^2+^	2.46	114Cys S–Zn^2+^	3.49
124Cys S–Zn^2+^	2.22	124Cys S–Zn^2+^	4.16
115Cys S–Zn^2+^	2.27	115Ile HB–Zn^2+^	3.45
119His ND–Zn^2+^	2.13	119His ND–Zn^2+^	4.08
114Cys S−124Cys S	2.99	114Cys S−124Cys S	5.82
115Cys S−119His ND	3.62	115Cys S−119His ND	7.24

## Discussion

In this study, we presented the development of novel Pb(II)-specific biosensors from existing genetic systems by engineering regulatory proteins and host cells. Although diverse bacterial cell-based biosensors have been reported for target metals and with corresponding genetic systems (Leveau and Lindow, 2002; Harms et al., 2006; Hynninen and Virta, 2009), heavy metals without corresponding genetic systems were excluded as targets of bacterial cell-based biosensors. In the case of Pb, the lead-resistant operon, *pbr* of *Cupriavidus metallidurans* CH34, was used for bacterial cell-based biosensors (Taghavi et al., 2009; Wei et al., 2014). Except for this previously described approach, there was no genetic system reported to date for obtaining a specific Pb(II) response. Therefore, the approaches described in this study to generate Pb(II)-specific biosensors are of great relevance, as they can represent an alternative bacterial cell-based biosensor for Pb(II) detection.

In fact, commercially available strains such as DH5α and BL21(DE3) could be used as host cells for biosensors. However, the BL21(DE3) was selected as a host cell because it possesses the IPTG-mediated overexpression systems, although the overexpression of regulatory genes was not necessary in this study. *E. coli* cell-based biosensors employing *znt*-operon showed strong responses toward Cd(II) and Hg(II), as reported previously (Yoon et al., 2016; Kang et al., 2018a). In addition, the recombinant ZntR was shown to effectively restore the metal-sensing properties of biosensors using *zntR*-deficient *E. coli* (*E. coli-zntR*) as host cells, with similar responses to those of the *znt*-operon-based system (data not shown). To evaluate the effects of metal ion channels (metal ion-translocating P-type ATPases), gene-deleted *E. coli* strains (*E. coli-zntR/copA, E. coli-zntR/zntA*, and *E. coli-zntR/copA/zntA*) were generated and used as host cells for the biosensors. Recombinant *zntR* WT was introduced as *zntAp::egfp* to the gene-deficient *E. coli* strains, and their metal selectivity was tested. However, this engineering approach was not suitable for evaluating the effects of the channels, since *zntA* deletion in the host cells induced eGFP overexpression even without metal exposure, thereby resulting in low induction coefficient values upon metal exposure. To overcome this problem, the initial cell density and duration for metal exposure were optimized. Briefly, the 0.05–0.30 of OD_600_ values were tested as initial cell density before metal ion exposure, and the fluorescence intensity was measured after 0.5–4 h. Since cell density of OD_600_ of 0.10 with 2 h exposure showed desirable results, it was selected as the optimal condition. Then, the metal selectivity of *E. coli* cell-based biosensors employing ZntR and *zntAp::egfp* as sensing and reporter domains, respectively, was changed by deleting the metal-exporting proteins (Figure 1). Deletion of *copA* and *zntA*, which encode CopA and ZntA, would impair the activity of Cu(I)- and Zn(II)-translocating P-type ATPases and consequently disrupt the metal homeostasis, resulting in the accumulation of metal ions in host cells (Sharma et al., 2000; Mitra and Sharma, 2001; Liu et al., 2006). The effects of CopA deletion weakened the responses of the biosensors toward Cd(II), whereas ZntA deletion enhanced their responses to Pb(II). In concordance with these results, weakened responses to Cd(II) and enhanced responses to Pb(II) were observed in both CopA- and ZntR-deleted strains (Figure 1D).

Next, the effects of genetic engineering of ZntR on target selectivity were investigated. Since the metal selectivity of the biosensors was determined by the target affinity of the regulatory proteins, they were selected as targets for genetic engineering to modulate metal selectivity (Ibáñez et al., 2015; Kang et al., 2018a; Lee et al., 2020). The *E. coli* cell-based biosensors investigated in this study employed *znt*-operon as a genetic system; thus, ZntR was the transcriptional regulator controlling the expression of the reporter gene (*egfp*). ZntR is one of the MerR family proteins known to unwind DNA and break two central base pairs upon target binding, thereby triggering transcriptional initiation (Heldwein and Brennan, 2001; Chen and He, 2004). Therefore, it was necessary to consider that mutations on its metal-binding site could not affect the function of the transcriptional factors. The MBL of the ZntR is composed of 12 amino acids, with three cysteines and one histidine, and two Zn(II) ions were placed on each loop region of ZntR (Changela et al., 2003; Pennella and Giedroc, 2005). As listed in Table 1, the numbers and positions of the cysteines were changed, and point mutations were introduced. Among the tested mutants, engineered ZntR Cys115Ser, H119R, and C141S showed stronger responses to Pb(II) rather than to Cd(II) (Figure 2). However, ZntR Cys115Ile showed the most marked changes concerning metal response compared with the other *E. coli-zntR/copA/zntA* systems. Although it showed enhanced response to Cd(II) compared with the WT, the response to Pb(II) was extremely increased to about 30-fold of the induction coefficient value. Nonetheless, this biosensor showed a broad specificity toward Pb(II), Cd(II), and Hg(II) at the tested concentration. To verify its specificity toward Pb(II), the biosensors were exposed to lower concentration ranges of the heavy metals (alone or in combination). Overall, the new biosensor responded only to Pb(II) at 0.1 μM and showed concentration-dependent responses from 0 to 100 nM of Pb(II) (Figures 3, 4). In addition, the other metal ions showed only marginal interference on signal acquisition at 10 nM (Figure 5), but no interference was observed at 5 nM of Cd(II) and Hg(II).

Finally, structural analysis was performed to better understand how the newly engineered biosensor achieved the observed superior selectivity and specificity toward Pb(II). The preference toward Pb(II) over Cd(II) was explained by the structural changes introduced around the MBLs of the ZntR (Figure 6). In particular, the 115Cys mutation disrupted the structural coordination with the zinc ions, thus resulting in decreased interaction with Cd(II) as compared with its originally stronger interaction. It was also noticed that the distances between the zinc ions and amino acids related to zinc binding, such as 114Cys, 115Cys, 124Cys, and 119His, were increased (Table 2), and the functions of the ZntRs as repressors were enhanced by the mutations (Figure 6). Although it was expected that conformational changes in the DNA-binding regions of ZntR could be responsible for the variations detected in the ZntR repressing activity, no significant structural differences around the DNA binding regions were observed. Thus, further investigations concerning the protein structures of the ZntRs are still warranted.

## Conclusion

**Figure 8:**
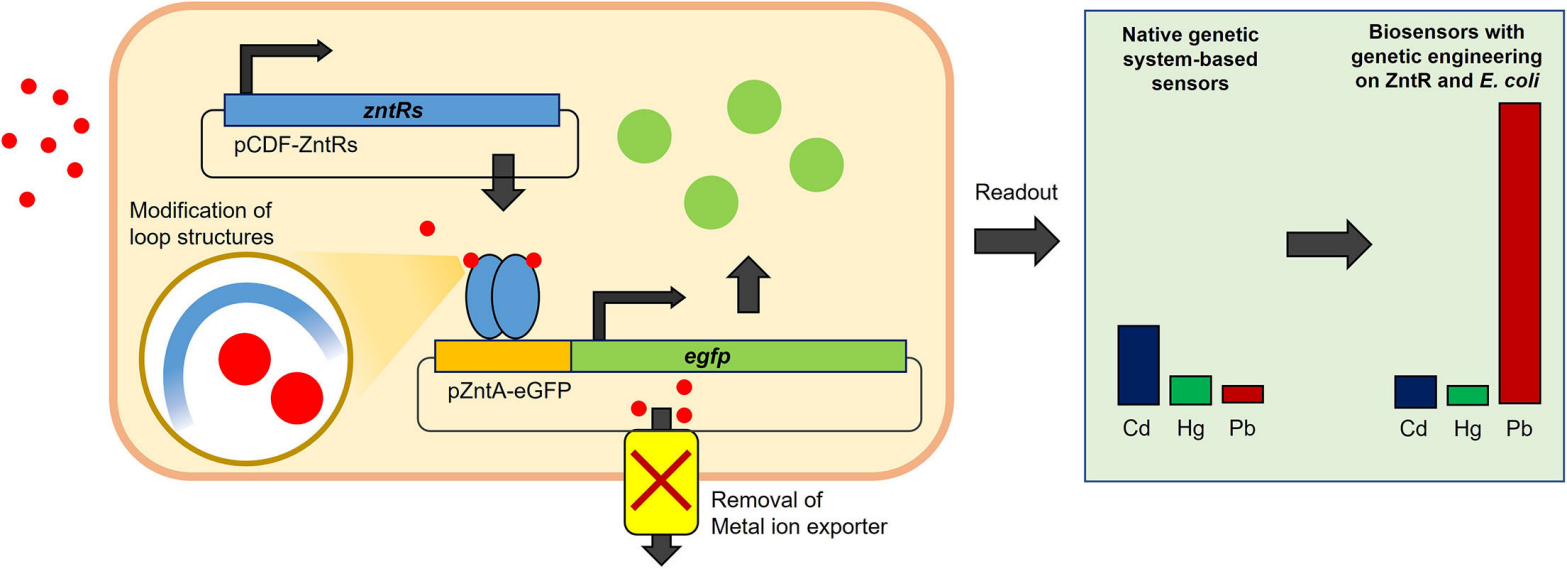
A schematic figure for the working mechanism of biosensor system. The regulatory protein, ZntR, repressed the expression of eGFP by binding on *zntA* promoter region without metal ions, while ZntR induced the expression of eGFP upon conformational change by metal ion binding. The *zntR* deleted *E. coli* BL21 harboring pCDF-ZntR WT and pZntA-eGFP showed responses in the order of Cd, Hg, and Pb, while the responding order and strength were modulated with modification of ZntR loop structure and with deleting metal exporting channel in host cells.

In this study, we described the generation of Pb(II)-specific biosensors, derived from existing genetic systems that can also be used as Cd(II) and Hg(II) biosensors. Briefly, the target selectivity of the regulatory protein, ZntR, was modulated by introducing point mutations on the metal-binding site, and the metal-exporting proteins were deleted from the host cells (Figure 8). The biosenor-based native *znt-*operon system shows the best response toward Cd(II), but its selectivity and specificity were shifted toward Pb(II) *via* genetic engineering. With the engineered ZntR C115I and *copA/zntA*-deficient *E. coli*, the novel biosensors possess superior selectivity and specificity to Pb(II), with a detection limit <5 nM. The Pb(II)-sensing capability of biosenors was sufficient to monitor the tolerance limit of lead in foods, which is set as 0.1–0.5 ppm (corresponding to ~1.2–6.0 μM) as defined by the Ministry of Food and Drug Safety in Korea. Thus, the Pb(II)-specific biosensor can be used as an alternative tool to monitor lead in foods and environmental samples. Although these bacterial cell-based biosensors were not actively applied to real samples, the engineering strategy used here may be of value to further enhance the capability and broaden the applications of cell-derived biosensors.

## Data Availability Statement

The original contributions presented in the study are included in the article/Supplementary Material, further inquiries can be directed to the corresponding author.

## Author Contributions

YY: conceptualization. YL and YJ: methodology, investigation, and data analysis. YY and B-GK: writing-original draft preparation. YY and GJ: writing-review and editing. All authors contributed to the article and approved the submitted version.

## Funding

This study was supported by Konkuk University in 2019 (to YY).

## Conflict of Interest

The authors declare that the research was conducted in the absence of any commercial or financial relationships that could be construed as a potential conflict of interest.

## Publisher's Note

All claims expressed in this article are solely those of the authors and do not necessarily represent those of their affiliated organizations, or those of the publisher, the editors and the reviewers. Any product that may be evaluated in this article, or claim that may be made by its manufacturer, is not guaranteed or endorsed by the publisher.
