# Supplementary material for: Design of Pb(II)-Specific E. coli-Based Biosensors by Engineering Regulatory Proteins and Host Cells
Source: Front Microbiol. 2022 May 20;13:881050. doi: 10.3389/fmicb.2022.881050 (PMC9164158; doi:10.3389/fmicb.2022.881050)
Supplement: Supplementary file 1 [file Data_Sheet_1.docx]

***Supplementary information for:***

**Development of new Pb(II)-specific *E. coli* cell-based biosensors by genetic engineering on regulatory proteins and host cells**

Yangwon Jeon^1^, Yejin Lee^1^, Geupil Jang^2^, Bong-Gyu Kim^3^, Youngdae Yoon^1,*^

^1^Department of Environmental Health Science, Konkuk University, Seoul 05029, Republic of Korea

^2^School of Biological Sciences and Technology, Chonnam National University, Gwangju 61186, Republic of Korea

^3^Department of Forest Resources, Gyeongsang National University of Science and Technology, Jinju 52725, Republic of Korea

***Corresponding author:**

**Youngdae Yoon,** Konkuk University, Korea

**E-mail:** yyoon21@gmail.com

**Online Resource 1. *E. coli* strains and plasmids lists**

|  | **Name** | **Genetic properties** | **reference** |
| --- | --- | --- | --- |
| *E. coli* | BL21(DE3)  BL21-*zntR*  BL21-*zntA*  BL21-*zntR/copA*  BL21-*zntR/copA/zntA* | F^-^ *ompT hsdS_B_*(r_B_^-^m_B_^-^)*gal dcm lon* (DE3)  BL21(DE3) *∆zntR*  BL21(DE3) *∆zntA*  BL21 (DE3) *∆zntR* and *∆copA*  BL21 (DE3) *∆zntR*, *∆copA and ∆zntA* | Stratagene  (Kang et al., 2018)  (Kang et al., 2018)  (Kang et al., 2018)  This study |
| Plasmids | pET-21(a)  pCDF-Duet  pZntA-eGFP  pCDF-ZntR  pCDF-ZntRs | pBR322 ori, Amp^r^  CloDE13 ori, Str^r^  pET-21(a) carrying *zntAp* from *E. coli* and *egfp* from pEGFP-N1  pCDF-Duet carrying *zntR* from *E. coli*  pCDF-Duet carrying *zntR* with point mutations | Novagen  Novagen  (Yoon et al., 2016)  (Yoon et al., 2018)  This study |

**Online Resource 2. Primer lists**

| **No.** | **Gene** | **Sequences (5’ to 3’)** | | |
| --- | --- | --- | --- | --- |
| 1 | zntAp | Forward  Reverse | AC**AGATCT**CGGCCTGCTACTTTGCC  CC**TCTAGA**GGGCTTTCTTGCCGTGAT | BglII  XbaI |
| 2 | zntR WT | Forward  Reverse | GTG**GGATCC**GATGTATCGCATTGGTGAG  AT**CTCGAG**TTACTTGTACAGCTCGTCCATGC | BamHI  XhoI |
| 3 | ZntR C115I | Forward  Reverse | GCCTGTTCTGGGACTGCTCATAGCAGT  ACTGCTATGAGCAGTCCCAGAACAGGC |  |
| 4 | ZntR C115S | Forward  Reverse | GCCTGTTCTGGGACTGCTCATAGCAGT  ACTGCTATGAGCAGTCCCAGAACAGGC |  |
| 5 | ZntR T117del | Forward  Reverse | GCCTGTTGTGGGGCTCATAGCAGTG  CACTGCTATGAGCCCCACAACAGGC |  |
| 6 | ZntR H119R | Forward  Reverse | GCCTGTATTGGGACTGCTCATAGCAGT  ACTGCTATGAGCAGTCCCAATACAGGC |  |
| 7 | ZntR C124S | Forward  Reverse | GCTCATAGCAGTGTTTATAGTTCGATTCTTGAAGCTC  GAGCTTCAAGAATCGAACTATAAACACTGCTATGAGC |  |
| 8 | ZntR C141S | Forward  Reverse | GCGTTAAGAGTGGTAGTTGACTCGACAAGC  GCTTGTCGAGTCAACTACCACTCTTAACGC |  |

^+^ The nucleotide sequences bold/underlined in No. 1 and 2 indicated the restriction enzyme sites.

^‡^ The pairs of primers for engineered ZntRs were complementary each other and used to introduce point mutations by site-directed mutagenesis.

**References**

Kang, Y., Lee, W., Jang, G., Kim, B.-G., and Yoon, Y. (2018). Modulating the sensing properties of Escherichia coli-based bioreporters for cadmium and mercury. *Applied microbiology and biotechnology* 102**,** 4863-4872.

Yoon, Y., Kang, Y., Lee, W., Oh, K.-C., Jang, G., and Kim, B.-G. (2018). Modulating the properties of metal-sensing whole-cell bioreporters by interfering with Escherichia coli metal homeostasis. *Journal of Microbiology and Biotechnology* 28**,** 323-329.

Yoon, Y., Kim, S., Chae, Y., Kang, Y., Lee, Y., Jeong, S.-W., and An, Y.-J. (2016). Use of tunable whole-cell bioreporters to assess bioavailable cadmium and remediation performance in soils. *PLoS One* 11**,** e0154506.
